# Reflex ROS1 IHC Screening with FISH Confirmation for Advanced Non-Small Cell Lung Cancer—A Cost-Efficient Strategy in a Public Healthcare System

**DOI:** 10.3390/curroncol28050284

**Published:** 2021-08-25

**Authors:** Maisam Makarem, Doreen A. Ezeife, Adam C. Smith, Janice J. N. Li, Jennifer H. Law, Ming-Sound Tsao, Natasha B. Leighl

**Affiliations:** 1 Princess Margaret Cancer Centre, University Health Network, Toronto, ON M5G 2C1, Canada; maisam.makarem2@uhn.ca (M.M.); adam.smith@uhn.ca (A.C.S.); janice.li@uhn.ca (J.J.N.L.); jennifer.law@uhn.ca (J.H.L.); ming.tsao@uhn.ca (M.-S.T.); 2 Department of Medicine, University of Toronto, Toronto, ON M5S 1A8, Canada; 3 Tom Baker Cancer Centre, University of Calgary, Calgary, AB T2N 4N2, Canada; doreen.ezeife@gmail.com; 4 Department of Laboratory Medicine and Pathobiology, University of Toronto, Toronto, ON M5G 2C4, Canada

**Keywords:** ROS1, IHC, reflex testing, NSCLC, cost, biomarker

## Abstract

*ROS1* rearrangements are identified in 1–2% of lung adenocarcinoma cases, and reflex testing is guideline-recommended. We developed a decision model for population-based *ROS1* testing from a Canadian public healthcare perspective to determine the strategy that optimized detection of true-positive (TP) cases while minimizing costs and turnaround time (TAT). Eight diagnostic strategies were compared, including reflex single gene testing via immunohistochemistry (IHC) screening, fluorescence in-situ hybridization (FISH), next-generation sequencing (NGS), and biomarker-informed (*EGFR/ALK/KRAS* wildtype) testing initiated by pathologists and clinician-initiated strategies. Reflex IHC screening with FISH confirmation of positive cases yielded the best results for TAT, TP detection rate, and cost. IHC screening saved CAD 1,000,000 versus reflex FISH testing. NGS was the costliest reflex strategy. Biomarker-informed testing was cost-efficient but delayed TAT. Clinician-initiated testing was the least costly but resulted in long TAT and missed TP cases, highlighting the importance of reflex testing. Thus, reflex IHC screening for ROS1 with FISH confirmation provides a cost-efficient strategy with short TAT and maximizes the number of TP cases detected.

## 1. Introduction

Advances in targeted therapies in lung cancer have changed the management and subsequent disease trajectory of lung cancer patients. There are now several genomic aberrations contributing to tumorigenesis that have shown improved patient outcomes with targeted therapy; these include mutations in *EGFR*, *BRAF*, and *MET* and rearrangements in *ALK*, *NTRK*, *RET*, and *ROS1*, among others [1]. *ROS1* gene rearrangements are present in 1–2% of all NSCLC [2,3] and are more commonly found in adenocarcinoma, younger patients, and never-smokers [3]. Crizotinib and entrectinib have been approved for the treatment of patients with *ROS1*-rearranged advanced non-squamous NSCLC [4,5] and have markedly improved outcomes in this patient population [6].

Methods for detecting *ROS1* rearrangements include immunohistochemistry (IHC) [7], fluorescence and other in-situ hybridization techniques (FISH), and next-generation sequencing (NGS). FISH is considered the validated standard, although NGS has emerged as an important alternative [8]. Guidelines from the International Association for the Study of Lung Cancer/College of American Pathologists, American Society of Clinical Oncology (ASCO), European Society for Medical Oncology (ESMO), and the Pan-Asian adapted ESMO Clinical Practice Guidelines all recommend that patients with advanced non-squamous NSCLC have reflex testing for *ROS1*, irrespective of clinical characteristics [1,9,10,11]. ASCO/Ontario Health [12], ESMO [10], NCCN [13], and other groups recommend targeted treatment for this subgroup of patients. 

However, not all jurisdictions have the same access to testing and targeted therapy. For example, in Ontario, Canada, public funding for *ROS1* testing and targeted treatment has only recently been approved [14]. While some jurisdictions can afford routine reflex NGS testing for multiple actionable markers in lung cancer samples, many cannot. The challenges of implementing *ROS1* testing as a population-based routine reflex diagnostic test include the low frequency of *ROS1* rearrangements, tissue requirements, and costs associated with testing. In publicly funded health care systems and those with lesser resources, identifying the most cost-efficient diagnostic testing strategy for single-gene tests like *ROS1* continues to be clinically relevant. Not all patients have funding or sufficient tissue for NGS, and not all patients are able to have repeat biopsies. 

We developed a population-based *ROS1* testing model for advanced non-squamous NSCLC to determine the most cost-efficient *ROS1* testing strategy from a Canadian public perspective that maximizes the detection of true positive cases (TP) while minimizing costs and turnaround time (TAT). We also included biomarker-informed testing after exclusion of *EGFR*-, *ALK*-, and *KRAS*-positive cases in order to explore potential cost savings and clinician-initiated testing.

## 2. Methods

### 2.1. Model Structure and Diagnostic Testing Strategies

**Figure 1:**
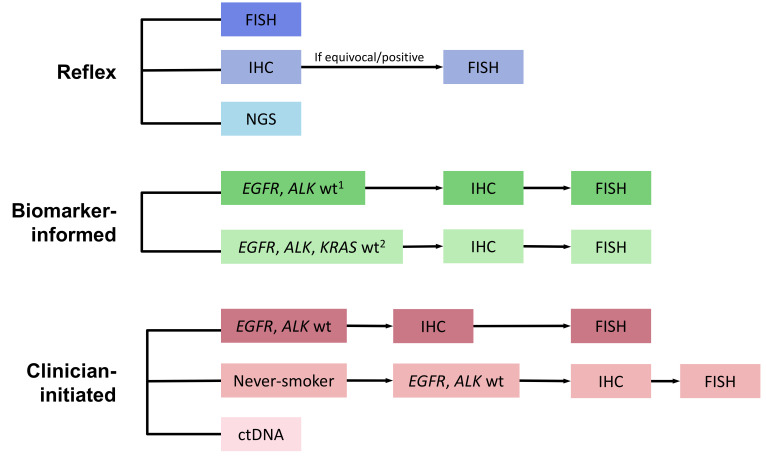
ROS-1 Testing Model Schema. ctDNA—circulating tumor DNA; FISH—Fluorescence in-situ hybridization; IHC—Immunohistochemistry; NGS—Next-generation sequencing; wt—wildtype. Footnotes: ^1^ If *EGFR*, *ALK*, or *KRAS* positive, then no further testing performed; wt—wildtype; ^2^ PCR-based sequencing.

A test-based decision modeling approach was developed using ROS1 IHC, FISH, and NGS sensitivity and specificity in addition to the prevalence of *ROS1* fusions. For IHC screening, positive predictive values (PPV) and negative predictive values (NPV) were calculated for each arm of the model to determine the proportion of patients who would first test positive after IHC screening and the subsequent proportion of patients who would undergo FISH confirmation (Figure 1, Tables S1–S9) [15]. Two models were developed with the calculated parameters to compare cost to true positive rates and cost to turnaround time of eight diagnostic testing strategies in patients with advanced non-squamous NSCLC (Figure S1). The target population was treatment-naïve patients with advanced non-squamous NSCLC in Ontario, Canada, estimated at 4000 new cases per year [16]. A total of 1000 patients were used for baseline value calculations. This analysis was conducted from the perspective of the Ontario health care system (University Health Network, Cancer Care Ontario, Toronto, ON, Canada), a publicly funded health care system [17].

Eight diagnostic strategies were compared, including reflex testing, serial testing informed by biomarker results, and clinician-initiated testing (Figure 1). Methods for reflex testing at the time of diagnosis in all non-squamous NSCLC cases included: (1) FISH; (2) IHC screening followed by FISH confirmation for positive cases [18,19,20], and (3) comprehensive NGS (DNA, RNA panel) [21]. The model assumed that actionable genomic alterations were mutually exclusive. Methods for serial testing informed by biomarker results included pathologist-initiated reflex testing of *EGFR*, *ALK*, and/or *KRAS* wild-type cases with IHC screening and FISH confirmation (hereafter described as “biomarker-informed”). Clinician-initiated testing strategies incorporated a selection of cases with *EGFR* and *ALK* wildtype, either limited or not limited to never-smokers. Finally, circulating tumor DNA (ctDNA) testing using a commercial assay in all patients was also included as a clinician-initiated strategy [22]. 

### 2.2. Model Assumptions

All patients were assumed to have accepted testing [23]. The model did not incorporate test failure rates or the option of repeat biopsies. The model accounted for the sensitivity and specificity of the *ROS1* assay using IHC (D4D6 clone (Cell Signaling) [17], FISH, NGS, and ctDNA. The sensitivities and specificities of the *EGFR*, *ALK*, and *KRAS* assays were not included in the model. 

### 2.3. Costs

All costs for diagnostic assays were estimated based on current University Health Network (UHN) Laboratory Medicine Program costs and provided in Canadian dollars (Table 1). The base case values included an estimate of reagent costs, sample processing, and handling costs and were based on expert opinion. Strategies involving serial testing included costs for *EGFR* and *KRAS* testing (as appropriate), based on validated PCR assays, and *ALK* testing using IHC [24,25]. Costs of ctDNA were based on estimated costs of a marketed commercial assay (e.g., Guardant360™, Guardant Health, Inc., Redwood City, CA, USA). Professional fees and administrative costs were not included. Costs were varied by 20% for sensitivity analysis unless otherwise indicated. Discounting was not applied, given the fixed time horizon included. Pricing, including for commercial liquid biopsy assays, is based on testing available to Ontario clinicians at the time the model was developed. 

### 2.4. Parameter Inputs

Parameter inputs used for the model are outlined in Table S1. The prevalence of *ROS1* gene rearrangements in patients with advanced NSCLC was not varied and was estimated at 1.5%. The frequencies of *EGFR*, *ALK*, and *KRAS* mutations in patients presenting with advanced non-squamous NSCLC were estimated as follows: *EGFR* mutation frequency was estimated at 17% [27], *ALK* at 3% [28], and *KRAS* at 25% [29]. We estimated the proportion of never-smokers in patients with advanced non-squamous NSCLC to be 20% (this was based on the mean of four studies that examined the proportion of never-smokers among patients presenting with NSCLC) [30,31,32,33]. The proportion of *EGFR*- and *ALK*-positive cases among never-smokers was estimated at 60% [34]. Lastly, the probability of *ROS1* fusions among *EGFR* and *ALK* wild-type never-smokers was estimated at 12% [35]. We assumed that *ROS1* fusions were exclusive of *EGFR*- and *ALK*-detected aberrations, such that all *ROS1*-positive cases would be detected among *EGFR* and *ALK* wild-type cases.

### 2.5. Outcomes 

The primary outcomes were result turnaround time (TAT) and the proportion of true-positive (TP) cases detected. The TAT (in days) for each diagnostic testing strategy included laboratory and sample processing time and was based on current UHN laboratory practices. The time to see an oncologist was based on the current Canadian Cancer Care Ontario recommendation of a 14-day target [36]. The effectiveness score for true-positive outcomes was set at 1 at terminal nodes. All other nodes were given a score of zero (this included true negatives, false positives, and false negatives). The model did not include final patient outcomes, i.e., quality-adjusted life years or treatment outcomes.

### 2.6. Base Case, Cost-Effectiveness, and Sensitivity Analysis

TreeAge Pro 2020 (TreeAge Software, Inc., Williamstown, MA, USA) was used to construct the decision trees and to complete base-case and cost- analyses (Figure S1). Base-case estimates were obtained, and variations around each were used for sensitivity analysis to assess the impact of variation around model inputs. Probabilities and assay sensitivities and specificities were varied as indicated in Table S1. Univariate sensitivity analysis was conducted on the parameter of cost and reported as a Tornado plot. The statistical software Matlab was used to generate the three-dimensional graph of TAT, TP detection rate, and cost for each testing strategy. 

## 3. Results

**Figure 2:**
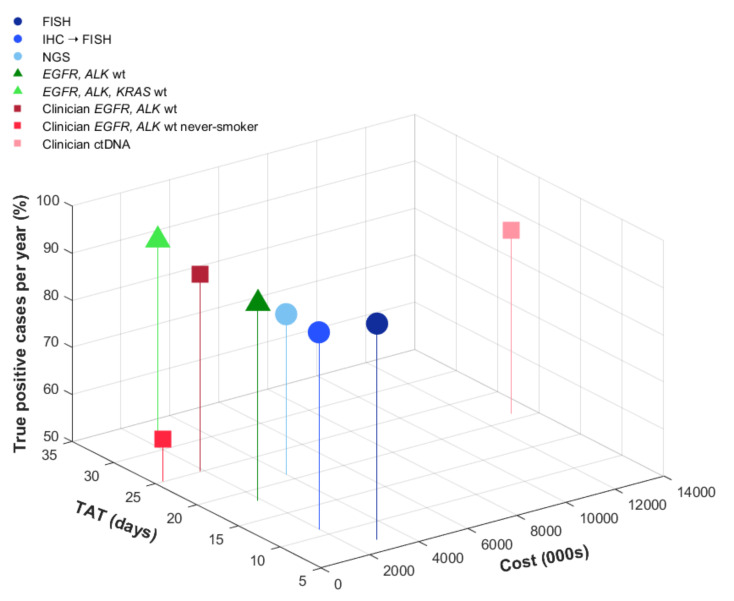
Cost, true-positive cases, and turnaround time. The graph shows base case analysis results of true-positive cases detected, turnaround time (in days), and cost (CAD). Reflex strategies initiated at diagnosis are shown in blue, biomarker-informed pathologist-initiated testing in green, and clinician-initiated strategies in red.

The rate of TP cases detected, TAT, and mean cost for each strategy are listed in Table 2. Although FISH was assumed to be the gold standard for this analysis, upfront NGS and IHC screening with FISH confirmation also yielded a high rate of TP cases. The strategy with the lowest yield of TP cases was clinician-initiated testing of patient samples that were *EGFR/ALK* wild-type in never smokers, missing 41% of TP cases (Figure 2). This strategy was also the least costly due to only a small proportion of patient samples undergoing testing, with a mean TAT of 25 days, longer than with pathologist-initiated reflex testing strategies. The other biomarker-informed testing strategies, i.e., testing of only *EGFR/ALK* wild-type samples, were also less costly due to a smaller number of cases undergoing testing. However, these all added significant length to resulting TAT. Incorporating *KRAS* testing into the biomarker-informed approach added cost and yielded the longest TAT (32 days, see Table 2).

**Figure 3:**
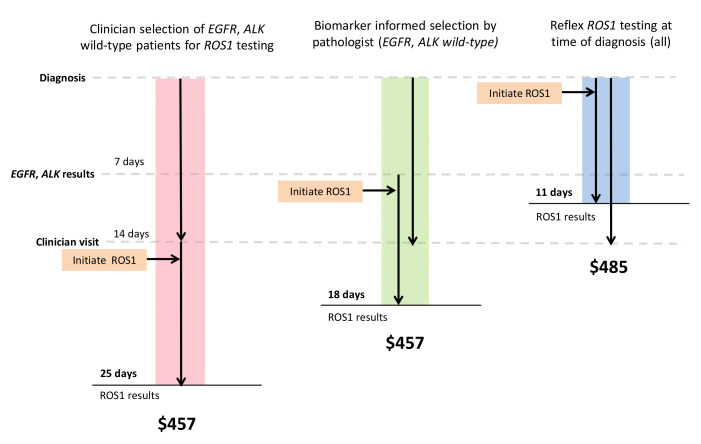
IHC screening with FISH confirmation most cost-efficient. Color bars represent the three strategies that are most cost-efficient in each arm of the model (clinician-initiated, biomarker-informed, and reflex testing). Black arrows demonstrate the time at which *ROS1* testing is initiated. Result turnaround time and estimated costs are labeled.

Balancing shorter TAT and higher TP rates, reflex IHC screening for ROS1 followed by FISH confirmation was the most cost-efficient strategy. This yielded an additional CAD 28 per case or CAD 112,000 for 4000 cases compared with molecular selection for *EGFR* and *ALK* wild-type cases (Table 2). Assuming 4000 cases tested per year, this approach would cost CAD 1,940,000, which would save CAD 1,020,000 compared to initial reflex testing with FISH alone. In addition, given the high specificity of ROS1 IHC, negative cases could be rapidly signed out with the shortest TAT, approximately 3 days, allowing results to be available at the time of clinician visit (Figure 3). The proportion of TP detected by this strategy would be similar to the biomarker informed testing strategies, assuming that *ROS1* fusions are mutually exclusive from *EGFR* mutations and *ALK* fusions (92%). 

More advanced sequencing technologies required longer TAT and had a higher cost. Among reflex testing strategies, NGS had the longest TAT (21 days) and was estimated to cost approximately CAD 4 million for 4000 cases. This approach would capture 84% (78–99%) of TP cases based on a sensitivity of 0.84 (base-value) and specificity of 0.99. The strategy of clinician-initiated ctDNA testing using commercial assays was most costly, estimated at CAD 13 million for 4000 cases (testing costs only). 

### Sensitivity Analysis

**Figure 4:**
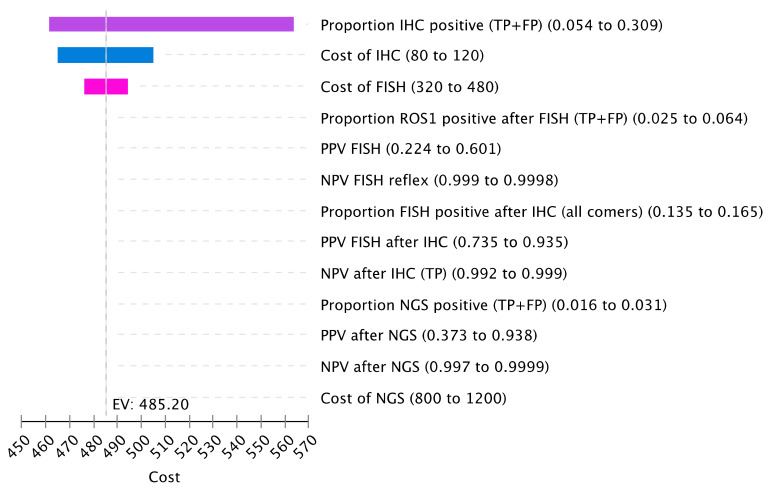
Sensitivity Analysis of Cost Drivers. The tornado diagram represents the one-way sensitivity analysis for cost. Select variables for cost were included for this plot. Input parameters are on the right with the listed variation. Variable definitions are listed in Table S9.

One-way sensitivity analysis was performed to test the influence of various parameters on cost. Reflex strategies were included in the analysis. The proportion of cases testing positive by IHC, which includes the sum of true positives and false-positive cases, had the greatest impact on cost (Figure 4). False-positive cases were also an important driver of cost in the case of the lower specificity of the ROS1 IHC assay. With a higher false-positive rate by IHC, more cases require FISH confirmation, increasing overall cost. The cost of the IHC and FISH assay also influenced overall costs. 

## 4. Discussion

Current biomarker testing strategies for patients with advanced NSCLC include testing for mutations and gene fusions that have approved targeted therapies with evidence of improved outcomes [1]. This includes aberrations in *EGFR*, *ALK*, expression of *PD-L1*, among several others [37]. Although more recent, the recommendation for *ROS1* testing is well-established, but the gold standard method to detect these gene rearrangements has been FISH testing, which is costly to apply broadly at a population level [19,20,38]. In this study, we used a decision-tree-based analytic model to compare the cost, result TAT, and TP detection rate between different diagnostic testing strategies to detect *ROS1* gene rearrangements. We show that reflex testing for *ROS1* fusions using IHC screening followed by FISH confirmation is the most cost-efficient approach. As part of reflex or routine testing, this strategy provides short TAT and a high proportion of TP detected as compared with other testing strategies. This is in large part due to the high sensitivity of the IHC assay, and concerns regarding lower specificity can be overcome with confirmation of positive cases by FISH testing [39]. 

Clinician-initiated testing is suboptimal for several reasons. Clinician-initiated strategies added 14 days to result TAT, based on current wait times to see a clinician from the diagnostic biopsy. We have previously shown that delays in molecular result TAT lead to delays in initiation of patient treatment and missed opportunities for targeted therapy [40]. The case for reflex testing is even more compelling when we consider that clinician-initiated testing is not systematic. We believe our strategy of clinicians requesting ROS-1 testing preferentially in patients that are never-smokers with *EGFR/ALK* wild-type tumors is common practice in systems without reflex testing. In our model, this approach missed up to 41% of TP cases and had prolonged TAT. Our model further highlights the importance of reflex testing, as lack of systematic testing will lead to less access to targeted therapy and inferior outcomes for patients. 

**Figure 5:**
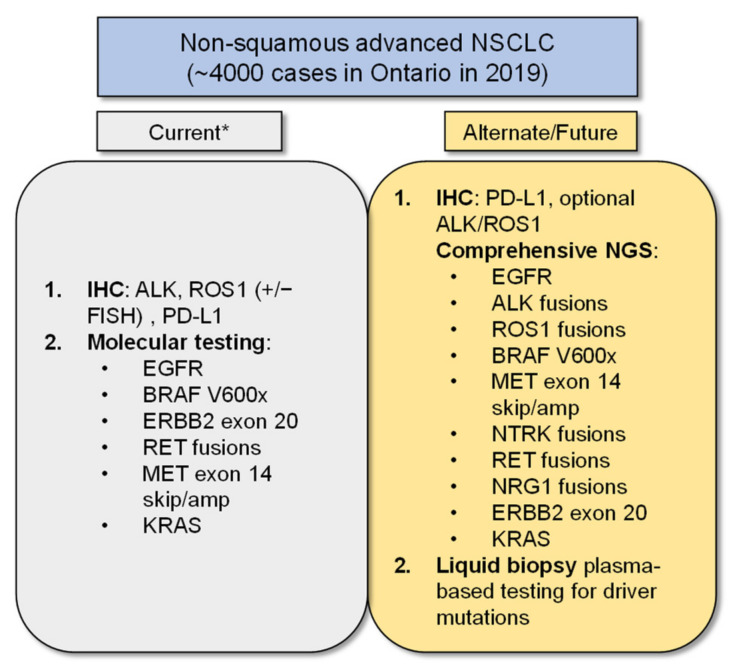
Ideal Biomarker Testing. The schematic shows current and future biomarker testing options for patients. Footnote: * At our institution, comprehensive NGS testing in addition to reflex PD-L1 IHC testing is now standard practice, but this has not been implemented across the province or country at the time this manuscript was written. This figure was adapted with modifications from [41].

The limitations of our study include the use of three single-gene assays for *EGFR*, *ALK*, and *ROS-1*, a current reality in our public system, in comparison to the growing practice of broader NGS testing for multiple targets now relevant in the care of advanced NSCLC patients (Figure 5, [41]). The costs of additional genomic targets in *BRAF*, *NTRK*, *RET*, *MET*, and emerging targets in *KRAS*, *ERBB2*, and *NRG*-1 were not included in our model as these are not yet funded as a standard of care in our jurisdiction. NGS testing platforms will become more widespread and will hopefully be incorporated as reflex testing around the world, in keeping with international guidelines [1], as they offer more complete genotyping, including the detection of uncommon genomic aberrations [42] and multiplexed target analysis, which reduces tissue consumption, all of which can impact clinical outcomes [43,44].

In our model, NGS was associated with the longest TAT and significant cost, although these costs may fall over time. In an *EGFR* mutant predominant population, upfront tissue NGS did not lead to significant increases in cost compared with standard of care molecular testing, and detected additional patients eligible for targeted therapy with short TAT [45]. In the United States, NGS has been shown to be cost-effective based on testing for seven or more genomic targets, although these results are not necessarily generalizable to other jurisdictions [46]. Similarly, our costs, based on the Ontario (Canada) public health system, may also have limited generalizability. For patients without comprehensive genomic tumor testing, those with small diagnostic samples, and those in jurisdictions with restricted health care funding, we believe that single-gene tests will remain relevant. However, it is hoped that NGS will become more accessible worldwide to allow more complete genotyping with a growing number of genomic targets needed for treatment decision-making for patients with advanced lung cancer. 

Liquid biopsy is an emerging and important technology to ensure that patients can have complete genotyping and has increasingly demonstrated clinical validity and utility in several prospective studies, especially in cases of insufficient tissue [26,47,48]. In our analysis, we used commercial list prices for this technology, resulting in routine ctDNA testing in all patients plus *EGFR/ALK* testing in tissue as the most expensive population-based testing strategy. Recent recommendations for plasma ctDNA testing include NGS-based platforms in treatment-naïve patients and in those who have developed drug resistance, now allowing a growing list of guideline-recommended targets to be assessed [49]. Although such platforms offer broad-based testing of known and unknown variants, non-NGS PCR-based ctDNA assays (either as a single gene or multiplexed), known to be highly sensitive for detecting a select few targetable alterations, may still offer a lower-cost alternative in resource-limited settings. However, liquid biopsy has been shown to help to avoid repeat tissue biopsy in patients with small samples, increase the identification of patients with actionable alterations, which may offset costs and offer short TAT [26,50]. Furthermore, it is expected that the costs of liquid biopsy technology will decrease over time, and the use of non-commercial assays or price negotiation may make this strategy more affordable in future. 

Another limitation of our analysis is that our clinical estimates of probabilities are based on published literature and expert opinion. A key concern with the use of ROS1 IHC screening relates to the variable specificity of current assays. With two antibody clones available and multiple ways of scoring the IHC assay (based on staining patterns, staining strengths, and H-score cutoffs) [1], confirmation by FISH (or NGS) remains necessary as part of the current standard. Validating the assay in accredited laboratories to ensure best practice continues to be of great importance in ensuring optimal patient outcomes. Lastly, the model does not take into account treatment outcomes, treatment costs, and the potential impact of false-negative results. 

## 5. Conclusions

In conclusion, we found that reflex ROS1 testing with IHC followed by FISH confirmation in positive cases is a cost-efficient approach with a high yield of true-positive cases and favorable result TAT (3 days or less for negative cases). Our study, which is conducted from the perspective of a publicly funded health care system (Ontario, Canada), underscores the importance of system-wide reflex testing for key biomarkers in lung cancer to optimize TAT and TP rates. Clinician-led strategies resulted in much longer TAT and the potential for missed cases. Biomarker-informed selection led to smaller testing volumes and therefore decreased costs, but significantly prolonged TAT. As the affordability of broad-spectrum NGS testing improves over time, we expect to see a shift from single-gene testing approaches to multiplexed approaches, even in jurisdictions with restricted healthcare funding. Until then, we believe our model demonstrates a feasible and affordable way to incorporate routine *ROS-1* testing in those jurisdictions that continue to struggle with the rising costs of cancer care, including genomic testing. 

## Figures and Tables

**Table 1:** Diagnostic test costs and turnaround time (TAT).

Cost (CAD) ^a^	Base Case	Range
EGFR+ALK	340	272–408
KRAS	200	400–600
ROS1 IHC ^b^	100	60–140
ROS1 FISH	400	320–480
NGS	1000	800–1200
ctDNA ^c^	3300	2640–3960
**TAT (Days)**		
Clinician time	14	11.2–16.8
EGFR, ALK	7	5.6–8.4
KRAS ^d^	21	16.8–25.2
ROS1 IHC	4	3.2–4.8
ROS1 FISH	7	5.6–8.4
NGS	21	16.8–25.2
ctDNA ^e^	7	4.8–7.2

^a^ Costs exclude administrative and professional fees. ^b^ ROS1 IHC costs were varied based on expert opinion. All other base case values were varied ±20%. ^c^ Based on estimated cost of a commercial assay. ^d^ TAT (in bold) estimated based on targeted NGS panel (including EGFR). ^e^ Based on TAT with Guardant360™ [26].

**Table 2:** Base case analysis of cost, turnaround time (TAT), and true-positive (TP) cases.

Testing Strategy	TP Proportion (%)	TAT ^a^ (days) Mean	TAT If *ROS1*+	Cost ^b^ (per Sample)	Cost (per 4000 Cases)	Incremental Cost for All vs. *EGFR*/*ALK* wt Only
**Reflex testing of all patients**						
FISH	96	7	7	740	2,960,000	1,132,000
IHC→FISH	92	7	11	485	1,940,000	112,000
NGS	84	21	21	1000	4,000,000	2,172,000
**Biomarker-informed**						
EGFR, ALK wt	92	11	18	457	1,828,000	-
EGFR, ALK, KRAS wt	92	24	32	622	2,488,000	660,000
**Clinical**						
EGFR, ALK wt	92	18	25	457	1,828,000	-
EGFR, ALK wt never-smokers	59	14	25	82	328,000	-
ctDNA	89	21	21	3300	13,200,000	11,372,000

wt—wildtype ^a^ This is the average TAT including *ROS1*-negative cases. The TAT for *ROS1*-positive includes time for FISH confirmation, *EGFR*, *ALK* ± *KRAS* testing. ^b^ Mean cost per case is listed in Canadian dollars and includes cost of *EGFR* and ALK testing. Testing strategies are bolded in grey rows.

## Data Availability

All data generated or analyzed during this study are included in this article (and its supplementary information files).

## Supplementary Materials

The following are available online at https://www.mdpi.com/article/10.3390/curroncol28050284/s1, Figure S1: Decision tree model of testing strategies, Table S1: Model Parameter inputs, Table S2: FISH test characteristics, Table S3: IHC screening (followed by FISH confirmation) test characteristics, Table S4: NGS test characteristics, Table S5: Biomarker informed selection of EGFR, ALK wildtype samples for ROS1 IHC screening and FISH confirmation, Table S6: Biomarker informed selection of EGFR, ALK, KRAS wildtype samples for ROS1 IHC screening and FISH confirmation, Table S7: Clinical selection of EGFR, ALK wildtype samples in never-smokers for ROS1 IHC screening and FISH confirmation, Table S8: ctDNA test characteristics, Table S9: Variable definitions.
