# Supplementary material for: Reflex ROS1 IHC Screening with FISH Confirmation for Advanced Non-Small Cell Lung Cancer—A Cost-Efficient Strategy in a Public Healthcare System
Source: Curr Oncol. 2021 Aug 25;28(5):3268–79. doi: 10.3390/curroncol28050284 (PMC8395515; doi:10.3390/curroncol28050284)
Supplement: Supplementary file 1 [file curroncol-28-00284-s001.zip › curroncol-1271801 suppl.pdf]

Supplementary Materials:

Reflex ROS1 IHC Screening with FISH Confirmation for Advanced Non-Small Cell Lung Cancer—A Cost-Efficient Strategy in a Public Healthcare System

Table of Contents

Figure S1. Decision tree model of testing strategies 2

Table S1. Model Parameter inputs. 3

Table S2. FISH test characteristics 5

Table S3. IHC screening (followed by FISH confirmation) test characteristics 6

Table S4. NGS test characteristics 7

Table S5 Biomarker informed selection of *EGFR, ALK* wildtype samples for ROS1 IHC screening and FISH confirmation 8

Table S6. Biomarker informed selection of *EGFR, ALK, KRAS* wildtype samples for ROS1 IHC screening and FISH confirmation 9

Table S7. Clinical selection of *EGFR, ALK* wildtype samples in never-smokers for ROS1 IHC screening and FISH confirmation 10

Table S8. ctDNA test characteristics..........

Table S9. Variable Definitions 12

References 14

Figure S1. Decision tree model of testing strategies

(Figure S1 attached separately)

Table S1. Model Parameter inputs.

Probabilities	Base-case	Variation		Source
		Lower Limit	Upper Limit	
**Assay Specificity and Sensitivity**				
FISH assay Sensitivity	0.96	0.95	0.99	[1]
FISH assay Specificity	0.96	0.95	0.99	[1]
NGS assay Sensitivity	0.84	0.78	0.99	[1]
NGS assay Specificity	0.99	0.98	0.99	[1, 2]
IHC assay Sensitivity	0.96	0.9	0.99	[3]lower limit based on expert opinion
IHC assay Specificity	0.9	0.7	0.96	[3]lower limit based on expert opinion
ctDNA Sensitivity	0.89	0.83	0.95	Guardant360
ctDNA Specificity	0.99	0.8	0.99	Guardant360
***ROS1* gene rearrangement prevalence**	0.015			[1, 4-8], expert opinion. Not varied
Diagnostic strategies				
***ROS1* FISH**				
Proportion of cohort who test positive	0.054	0.025	0.064	
PPV FISH	0.268	0.224	0.601	
NPV FISH	0.999	0.999	0.9998	
**ROS1 IHC then FISH**				
Proportion testing positive after IHC	0.113	0.135	0.165	
Proportion of cohort who test positive after FISH confirmation	0.157	0.135	0.165	
PPV after IHC then FISH	0.778	0.735	0.935	
NPV after IHC then FISH	0.994	0.992	0.999	
***EGFR, ALK* wildtype**				
Proportion of cohort testing EGFR, ALK positive	0.20			[9, 10]
Proportion testing positive after IHC	0.116	0.058	0.311	
Proportion testing positive by FISH after IHC	0.183	0.162	0.190	
PPV after IHC then FISH	0.815	0.777	0.948	
NPV after IHC then FISH	0.992	0.990	0.998	
***EGFR, ALK, KRAS* wildtype**				
Proportion of cohort testing EGFR, ALK, KRAS positive	0.45			[9-11]
Proportion testing positive after IHC	0.123	0.066	0.316	
Proportion testing positive by FISH after IHC	0.235	0.218	0.241	
PPV after IHC then FISH	0.866	0.836	0.964	
NPV after IHC then FISH	0.989	0.986	0.997	
***EGFR, ALK* wildtype never-smokers**				
Proportion of never smokers	0.20			[12-15]
Proportion of EGFR and ALK positive cases among never-smokers	0.60			[16]

Proportion of ROS1 positive cases among never-smokers, EGFR, ALK wild-type cases	0.12			17
Proportion testing positive after IHC	0.203	0.154	0.372	
Proportion testing positive by FISH after IHC	0.562	0.560	0.566	
PPV after IHC then FISH	0.969	0.961	0.992	
NPV after IHC then FISH	0.948	0.936	0.987	
**NGS**				
Proportion who test positive after NGS	0.022	0.016	0.031	
PPV NGS	0.561	0.373	0.938	
NPV NGS	0.998	0.997	0.9999	
**ctDNA**				
Proportion who test positive after ctDNA	0.023	0.015	0.209	
PPV ctDNA	0.575	0.059	0.935	
NPV ctDNA	0.998	0.997	0.999	

Table S2. FISH test characteristics

	Disease positive (D+)	Disease negative (D-)	Total
**Test Positive (T+)**	A - True Positives (TP)	B- False Positives (FP)	A+B (TP+FP)
**Test Negative (T-)**	C - False negatives (FN)	D -True Negatives (TN)	C+D (FN +TN)
	A+C	B+ D	Cohort (A+B+C+D)

	Probabilities	Variation	
**Assay Specificity and Sensitivity**		**Lower Limit**	**Upper Limit**
FISH assay Sensitivity	0.96	0.95	0.99
FISH assay Specificity	0.96	0.95	0.99
ROS1 gene prevalence	0.015		

		Base Case	Variation	
**Population (Cohort)**	Cohort*	1000.00		
**Disease positive (D+)**	ROS1 positive cases**	15.00		
**Disease negative (D-)**	ROS1 negative cases	985.00		
**A, TP**	Sensitivity X (D+)	14.40	14.25	14.85
**C, FN**	(D+) - TP	0.60	0.75	0.15
**D, TN**	Specificity X (D-)	945.60	935.75	975.15
**B, FP**	(D-) - TN	39.40	49.25	9.85
**Total Test positive (T+)**	TP+ FP (A+B)	53.80	63.50	24.70
**Total Test negative (T -)**	TN + FN (C+D)	946.20	936.50	975.30

		Base Case	Variation	
**PPV**	A/(A+B)	0.268	0.224	0.601
**NPV**	D/(C+D)	0.999	0.999	0.9998
**Proportion testing positive**	Total T+/Cohort	0.054	0.064	0.025

*Assuming1000 patients with advanced non-squamous NSCLC

** Based on ROS1 fusion prevalence

Table S3. IHC screening (followed by FISH confirmation) test characteristics

	Probabilities	Variation	
**Assay Specificity and Sensitivity**		**Lower Limit**	**Upper Limit**
FISH assay Sensitivity	0.96	0.95	0.99
FISH assay Specificity	0.96	0.95	0.99
IHC assay Sensitivity	0.96	0.90	0.99
IHC assay Specificity	0.9	0.70	0.96
ROS1 gene prevalence	0.015		

		Base Case, IHC first	Variation		Base Case, IHC then FISH	Variation	
**Population (Cohort)**	Cohort*	1000.00			112.90		
**Disease positive (D+)**	ROS1 positive cases**	15.00			14.40		
**Disease negative (D-)**	ROS1 negative cases	985.00			98.50		
**A, TP**	Sensitivity X (D+)	14.40	13.50	14.85	13.82	13.68	14.26
**C, FN**	(D+) - TP	0.60	1.50	0.15	0.58	0.72	0.14
**D, TN**	Specificity X (D-)	886.50	689.50	945.60	94.56	93.58	97.52
**B, FP**	(D-) - TN	98.50	295.50	39.40	3.94	4.93	0.98
**Total Test positive (T+)**	TP+ FP (A+B)	112.90	309.00	54.25	17.76	18.61	15.24
**Total Test negative (T -)**	TN + FN (C+D)	887.10	691.00	945.75	95.14	94.30	97.66

IHC		Base Case	Variation	
**PPV**	A/(A+B)	0.128	0.044	0.274
**NPV**	D/(C+D)	0.999	0.998	0.9998
**Proportion testing positive**	Total T+/Cohort	0.113	0.309	0.054

FISH after IHC		Base Case	Variation	
**PPV**	A/(A+B)	0.778	0.735	0.935
**NPV**	D/(C+D)	0.994	0.992	0.999
**Proportion testing positive**	Total T+/Cohort	0.157	0.165	0.135

*Assuming1000 patients with advanced non-squamous NSCLC

** Based on ROS1 fusion prevalence

Table S4. NGS test characteristics

	Probabilities	Variation	
**Assay Specificity and Sensitivity**		**Lower Limit**	**Upper Limit**
NGS assay Sensitivity	0.84	0.78	0.999
NGS assay Specificity	0.99	0.98	0.999
ROS1 gene prevalence	0.015		

		Base Case	Variation	
**Population (Cohort)**	Cohort*	1000.00		
**Disease positive (D+)**	ROS1 positive cases**	15.00		
**Disease negative (D-)**	ROS1 negative cases	985.00		
**A, TP**	Sensitivity X (D+)	12.60	11.70	14.99
**C, FN**	(D+) - TP	2.40	3.30	0.02
**D, TN**	Specificity X (D-)	975.15	965.30	984.02
**B, FP**	(D-) - TN	9.85	19.70	0.99
**Total Test positive (T+)**	TP+ FP (A+B)	22.45	31.40	15.97
**Total Test negative (T -)**	TN + FN (C+D)	977.55	968.60	984.03

		Base Case	Variation	
**PPV**	A/(A+B)	0.561	0.373	0.938
**NPV**	D/(C+D)	0.998	0.997	0.99998
**Proportion testing positive**	Total T+/Cohort	0.022	0.031	0.016

*Assuming1000 patients with advanced non-squamous NSCLC

** Based on ROS1 fusion prevalence

Table S5 Biomarker informed selection of *EGFR, ALK* wildtype samples for ROS1 IHC screening and FISH confirmation

	**Probabilities**	**Variation**	
**Assay Specificity and Sensitivity**		**Lower Limit**	**Upper Limit**
FISH assay Sensitivity	0.96	0.95	0.99
FISH assay Specificity	0.96	0.95	0.99
IHC assay Sensitivity	0.96	0.90	0.99
IHC assay Specificity	0.9	0.70	0.96
Proportion of cohort testing EGFR, ALK positive	0.2		

		**Base Case, IHC first**	**Variation**		**Base Case, after FISH**	**Variation**	
**Population (Cohort)**	Cohort*	800.00			92.90		
**Disease positive (D+)**	ROS1 positive cases	15.00			14.40		
**Disease negative (D-)**	ROS1 negative cases	785.00			78.50		
**A, TP**	Sensitivity X (D+)	14.40	13.50	14.85	13.82	13.68	14.26
**C, FN**	(D+) - TP	0.60	1.50	0.15	0.58	0.72	0.14
**D, TN**	Specificity X (D-)	706.50	549.50	753.60	75.36	74.58	77.72
**B, FP**	(D-) - TN	78.50	235.50	31.40	3.14	3.93	0.78
**Total Test positive (T+)**	TP+ FP (A+B)	92.90	249.00	46.25	16.96	17.61	15.04
**Total Test negative (T -)**	TN + FN (C+D)	707.10	551.00	753.75	75.94	75.30	77.86

IHC first		**Base Case**	**Variation**	
**PPV**	A/(A+B)	0.155	0.054	0.321
**NPV**	D/(C+D)	0.999	0.997	0.9998
**Proportion testing positive**	Total T+/Cohort	0.116	0.311	0.058

FISH after IHC		**Base Case**	**Variation**	
**PPV**	A/(A+B)	0.815	0.777	0.948
**NPV**	D/(C+D)	0.992	0.990	0.998
**Proportion testing positive**	Total T+/Cohort	0.183	0.190	0.162

*Based on proportion of patients who test positive for *EGFR, ALK* in a 1000 patient cohort

Table S6. Biomarker informed selection of *EGFR, ALK, KRAS* wildtype samples for ROS1 IHC screening and FISH confirmation

	Probabilities	Variation	
**Assay Specificity and Sensitivity**		**Lower Limit**	**Upper Limit**
FISH assay Sensitivity	0.96	0.95	0.99
FISH assay Specificity	0.96	0.95	0.99
IHC assay Sensitivity	0.96	0.90	0.99
IHC assay Specificity	0.9	0.70	0.96
Proportion of cohort testing *EGFR, ALK* positive	0.2		
Proportion of cohort testing *EGFR, ALK, KRAS* negative	0.55		

		**Base Case, IHC first**	**Variation**		**Base Case, after FISH**	**Variation**	
**Population (Cohort)**	Cohort*	550.00			67.90		
**Disease positive (D+)**	ROS1 positive cases**	15.00			14.40		
**Disease negative (D-)**	ROS1 negative cases	535.00			53.50		
**A, TP**	Sensitivity X (D+)	14.40	13.50	14.85	13.82	13.68	14.26
**C, FN**	(D+) - TP	0.60	1.50	0.15	0.58	0.72	0.14
**D, TN**	Specificity X (D-)	481.50	374.50	513.60	51.36	50.83	52.97
**B, FP**	(D-) - TN	53.50	160.50	21.40	2.14	2.68	0.54
**Total Test positive (T+)**	TP+ FP (A+B)	67.90	174.00	36.25	15.96	16.36	14.79
**Total Test negative (T -)**	TN + FN (C+D)	482.10	376.00	513.75	51.94	51.55	53.11

IHC first		**Base Case**	**Variation**	
**PPV**	A/(A+B)	0.212	0.078	0.410
**NPV**	D/(C+D)	0.999	0.996	0.9997
**Proportion testing positive**	Total T+/Cohort	0.123	0.316	0.066

FISH after IHC		**Base Case**	**Variation**	
**PPV**	A/(A+B)	0.866	0.836	0.964
**NPV**	D/(C+D)	0.989	0.986	0.997
**Proportion testing positive**	Total T+/Cohort	0.235	0.241	0.218

*Cohort of patients testing *EGFR, ALK, KRAS* wildtype

Table S7. Clinical selection of *EGFR, ALK* wildtype samples in never-smokers for ROS1 IHC screening and FISH confirmation

	Probabilities	Variation	
**Assay Specificity and Sensitivity**		**Lower Limit**	**Upper Limit**
FISH assay Sensitivity	0.96	0.95	0.99
FISH assay Specificity	0.96	0.95	0.99
IHC assay Sensitivity	0.96	0.90	0.99
IHC assay Specificity	0.9	0.70	0.96
Proportion of never smokers	0.2		
Proportion of *EGFR, ALK* positive among never-smokers	0.6		
Proportion of *ROS1* positive cases among never-smokers, *EGFR, ALK* negative cases	0.12		

		**Base Case, IHC first**	**Variation**		**Base Case, after FISH**	**Variation**	
**Population (Cohort)**	Cohort*	80.00			16.26		
**Disease positive (D+)**	ROS1 positive cases**	9.60			9.22		
**Disease negative (D-)**	ROS1 negative cases	70.40			7.04		
**A, TP**	Sensitivity X (D+)	9.22	8.64	9.50	8.85	8.76	9.12
**C, FN**	(D+) - TP	0.38	0.96	0.10	0.37	0.46	0.09
**D, TN**	Specificity X (D-)	63.36	49.28	67.58	6.76	6.69	6.97
**B, FP**	(D-) - TN	7.04	21.12	2.82	0.28	0.35	0.07
**Total Test positive (T+)**	TP+ FP (A+B)	16.26	29.76	12.32	9.13	9.11	9.19
**Total Test negative (T -)**	TN + FN (C+D)	63.74	50.24	67.68	7.13	7.15	7.06

IHC first		**Base Case**	**Variation**	
**PPV**	A/(A+B)	0.567	0.290	0.771
**NPV**	D/(C+D)	0.994	0.981	0.9986
**Proportion testing positive**	Total T+/Cohort	0.203	0.372	0.154

FISH after IHC		**Base Case**	**Variation**	
**PPV**	A/(A+B)	0.969	0.961	0.992
**NPV**	D/(C+D)	0.948	0.936	0.987
**Proportion testing positive**	Total T+/Cohort	0.562	0.560	0.566

*Based on proportion of *EGFR, ALK* wildtype never-smokers

**Based on estimated *ROS1* fusion prevalence among *EGFR, ALK* wildtype never-smokers

Table S8. ctDNA test characteristics

	Probabilities	Variation	
**Assay Specificity and Sensitivity**		**Lower Limit**	**Upper Limit**
ctDNA Sensitivity	0.89	0.83	0.95
ctDNA Specificity	0.99	0.80	1.00
ROS1 gene prevalence	0.015		

		Base Case	Variation	
**Population (Cohort)**	Cohort*	1000.00		
**Disease positive (D+)**	ROS1 positive cases**	15.00		
**Disease negative (D-)**	ROS1 negative cases	985.00		
**A, TP**	Sensitivity X (D+)	13.35	12.45	14.25
**C, FN**	(D+) - TP	1.65	2.55	0.75
**D, TN**	Specificity X (D-)	975.15	788.00	984.02
**B, FP**	(D-) - TN	9.85	197.00	0.99
**Total Test positive (T+)**	TP+ FP (A+B)	23.20	209.45	15.24
**Total Test negative (T -)**	TN + FN (C+D)	976.80	790.55	984.77

		Base Case	Variation	
**PPV**	A/(A+B)	0.575	0.059	0.935
**NPV**	D/(C+D)	0.998	0.997	0.9992
**Proportion testing positive**	Total T+/Cohort	0.023	0.209	0.015

*Assuming 1000 patients with advanced non-squamous NSCLC

**Based on ROS1 fusion prevalence

Table S9. Variable Definitions

Name	Description	Root Definition	Low	High
cCTDNA	Cost of ctDNA	3300	0.2	0.2
cEGFRALK	Cost of EGFR + ALK	340	0.2	0.2
cFISH	Cost of FISH	400	0.2	0.2
cIHC	Cost of IHC	100	0.2	0.2
cKRAS	Cost of KRAS testing	200	0.2	0.2
cNGS	Cost of NGS	1000	0.2	0.2
cNo_Rebiopsy	Cost of no re-biopsy	120	0	120
cRe_Biopsy	Cost of re-biopsy	1500	0	1500
NPV_FISH	NPV FISH reflex	0.999	0.999	0.9998
NPVafterctDNA	NPV after ctDNA testing	0.998	0.997	0.999
NPVafterIHC	NPV after IHC (TP)	0.994	0.992	0.999
NPVafterIHCEGFRALKKRASneg	NPV after EGFR, ALK, KRAS	0.989	0.986	0.997
NPVafterIHCEGFRALKneg	NPV after EGFR, ALK	0.992	0.99	0.998
NPVNeversmoker	NPV after Never-smoker, EGFR, ALK	0.948	0.963	0.987
NPVNGS	NPV after NGS	0.998	0.997	0.9999
pEGFRALKKRASpos	Proportion EGFR, ALK, KRAS positive	0.45	0	0.45
pEGFRALKpos	Proportion EGFR, ALK positive	0.2	0	0.2
pEGFRALKposamongneversmokers	Proportion Never-smokers, EGFR, ALK positive	0.6	0	0.6
pFISHposafterIHCEGFRALK	Proportion FISH positive after IHC (EGFR, ALK wt)	0.183	0.162	0.19
pFISHposafterIHCEGFRALKKRAS	Proportion FISH positive after IHC (EGFR, ALK, KRAS wt)	0.235	0.218	0.241
pFISHposafterIHCEGFRALKwtneversmoker	Proportion FISH positive after IHC (Never-smoker,EGFR, ALK wt)	0.562	0.56	0.566
pFISHpositiveafterIHC	Proportion FISH positive after IHC (all comers)	0.157	0.135	0.165
pIHCposafterEGFRALK	Proportion IHC positive if EGFR ALK wt	0.116	0.058	0.311
pIHCposafterEGFRALKKRAS	Proportion IHC positive if EGFR ALK, KRAS wt	0.123	0.066	0.316
pIHCposamongEGFRALKwtneversmokers	Proportion IHC positive if Never-smoker, EGFR ALK wt	0.203	0.154	0.372
pNeversmoker	Proportion of neversmokers	0.2	0	0.2
PPV_FISH	PPV FISH	0.268	0.224	0.601
PPVafterctDNA	PPV after ctDNA testing	0.575	0.059	0.935

PPVafterIHC	PPV FISH after IHC	0.778	0.735	0.935
PPVafterIHCEGFRALK	PPV FISH after IHC (EGFR, ALK wt)	0.815	0.777	0.948
PPVafterIHCEGFRALKKRAS	PPV FISH after IHC (EGFR, ALK, KRAS wt)	0.866	0.836	0.964
PPVneversmoker	PPV FISH after IHC (Never-smokers, EGFR, ALK wt)	0.969	0.961	0.992
PPVNGS	PPV after NGS	0.561	0.373	0.938
pTestpositivectDNA	Proportion ROS1 positive with ctDNA (TP+FP)	0.0232	0.015	0.209
pTestpositiveFISH	Proportion ROS1 positive after FISH (TP+FP)	0.0538	0.025	0.064
pTestpositiveIHC	Proportion IHC positive (TP+FP)	0.113	0.054	0.309
pTestpositiveNGS	Proportion NGS positive (TP+FP)	0.022	0.016	0.031
TATClinician	Time to clinician visit	14	0.2	0.2
TATctDNA	Turnaround time ctDNA	7	0.2	0.2
TATEGFRALK	Turnaround time for EGFR, ALK testing	7	0.2	0.2
TATFISH	Turnaround time FISH	7	0.2	0.2
TATIHC	Turnaround time ROS1 IHC	4	0.2	0.2
TATKRAS	Turnaround time KRAS	21	0.2	0.2
TATNGS	Turnaround time NGS	21	0.2	0.2

References

1. Davies KD, Le AT, Sheren J, et al. Comparison of Molecular Testing Modalities for Detection of ROS1 Rearrangements in a Cohort of Positive Patient Samples. Research Support, N.I.H., Extramural. *J Thorac Oncol*. Oct 2018;13(10):1474-1482. doi:10.1016/j.jtho.2018.05.041
2. Velizheva NP, Rechsteiner MP, Valtcheva N, et al. Targeted next-generation-sequencing for reliable detection of targetable rearrangements in lung adenocarcinoma-a single center retrospective study. *Pathol Res Pract*. Apr 2018;214(4):572-578. doi:10.1016/j.prp.2018.02.001
3. Lindeman NI, Cagle PT, Aisner DL, et al. Updated Molecular Testing Guideline for the Selection of Lung Cancer Patients for Treatment With Targeted Tyrosine Kinase Inhibitors: Guideline From the College of American Pathologists, the International Association for the Study of Lung Cancer, and the Association for Molecular Pathology. *J Thorac Oncol*. 03 2018;13(3):323-358. doi:10.1016/j.jtho.2017.12.001
4. Bergethon K, Shaw AT, Ou SH, et al. ROS1 rearrangements define a unique molecular class of lung cancers. Research Support, N.I.H., Extramural Research Support, Non-U.S. Gov't. *J Clin Oncol*. Mar 10 2012;30(8):863-70. doi:10.1200/jco.2011.35.6345
5. Go H, Kim DW, Kim D, et al. Clinicopathologic analysis of ROS1-rearranged non-small-cell lung cancer and proposal of a diagnostic algorithm. *J Thorac Oncol*. Nov 2013;8(11):1445-50. doi:10.1097/JTO.0b013e3182a4dd6e
6. Takeuchi K, Soda M, Togashi Y, et al. RET, ROS1 and ALK fusions in lung cancer. Research Support, Non-U.S. Gov't. *Nat Med*. Feb 12 2012;18(3):378-81. doi:10.1038/nm.2658
7. Yoshida A, Kohno T, Tsuta K, et al. ROS1-rearranged lung cancer: a clinicopathologic and molecular study of 15 surgical cases. Research Support, Non-U.S. Gov't. *Am J Surg Pathol*. Apr 2013;37(4):554-62. doi:10.1097/PAS.0b013e3182758fe6
8. Rimkunas VM, Crosby KE, Li D, et al. Analysis of receptor tyrosine kinase ROS1-positive tumors in non-small cell lung cancer: identification of a FIG-ROS1 fusion. *Clin Cancer Res*. Aug 15 2012;18(16):4449-57. doi:10.1158/1078-0432.ccr-11-3351
9. Ellis PM, Verma S, Sehdev S, Younus J, Leighl NB. Challenges to implementation of an epidermal growth factor receptor testing strategy for non-small-cell lung cancer in a publicly funded health care system. *J Thorac Oncol*. Sep 2013;8(9):1136-41. doi:10.1097/JTO.0b013e31829f6a43
10. Hallberg B, Palmer RH. Mechanistic insight into ALK receptor tyrosine kinase in human cancer biology. *Nat Rev Cancer*. Oct 2013;13(10):685-700. doi:10.1038/nrc3580
11. Kris MG, Johnson BE, Kwiatkowski DJ, et al. Identification of driver mutations in tumor specimens from 1,000 patients with lung adenocarcinoma: The NCI's Lung Cancer Mutation Consortium (LCMC). *Journal of Clinical Oncology*. 2011/05/20 2011;29(15_suppl):CRA7506-CRA7506. doi:10.1200/jco.2011.29.15_suppl.cra7506

12. Cho J, Choi SM, Lee J, et al. Proportion and clinical features of never-smokers with non-small cell lung cancer. *Chinese Journal of Cancer*. 2017/02/08 2017;36(1):20. doi:10.1186/s40880-017-0187-6
13. Pelosof L, Ahn C, Gao A, et al. Proportion of Never-Smoker Non-Small Cell Lung Cancer Patients at Three Diverse Institutions. *Journal of the National Cancer Institute*. 2017;109(7):djw295. doi:10.1093/jnci/djw295
14. Santoro IL, Ramos RP, Franceschini J, Jamnik S, Fernandes ALG. Non-small cell lung cancer in never smokers: a clinical entity to be identified. *Clinics (Sao Paulo, Brazil)*. 2011;66(11):1873-1877. doi:10.1590/s1807-59322011001100005
15. Dias M, Linhas R, Campainha S, Conde S, Barroso A. Lung cancer in never-smokers – what are the differences? *Acta Oncologica*. 2017/07/03 2017;56(7):931-935. doi:10.1080/0284186X.2017.1287944
16. Korpanty GJ, Kamel-Reid S, Pintilie M, et al. Lung cancer in never smokers from the Princess Margaret Cancer Centre. *Oncotarget*. Apr 2018;9(32):22559-22570. doi:10.18632/oncotarget.25176
17. Marchetti A, Barberis M, Di Lorito A, et al. ROS1 Gene Fusion in Advanced Lung Cancer in Women: A Systematic Analysis, Review of the Literature, and Diagnostic Algorithm. *JCO Precision Oncology*. 2017/11/01 2017;(1):1-9. doi:10.1200/PO.16.00010
