# Supplementary figures and images for: Reflex ROS1 IHC Screening with FISH Confirmation for Advanced Non-Small Cell Lung Cancer—A Cost-Efficient Strategy in a Public Healthcare System
Source: Curr Oncol. 2021 Aug 25;28(5):3268–79. doi: 10.3390/curroncol28050284 (PMC8395515; doi:10.3390/curroncol28050284)

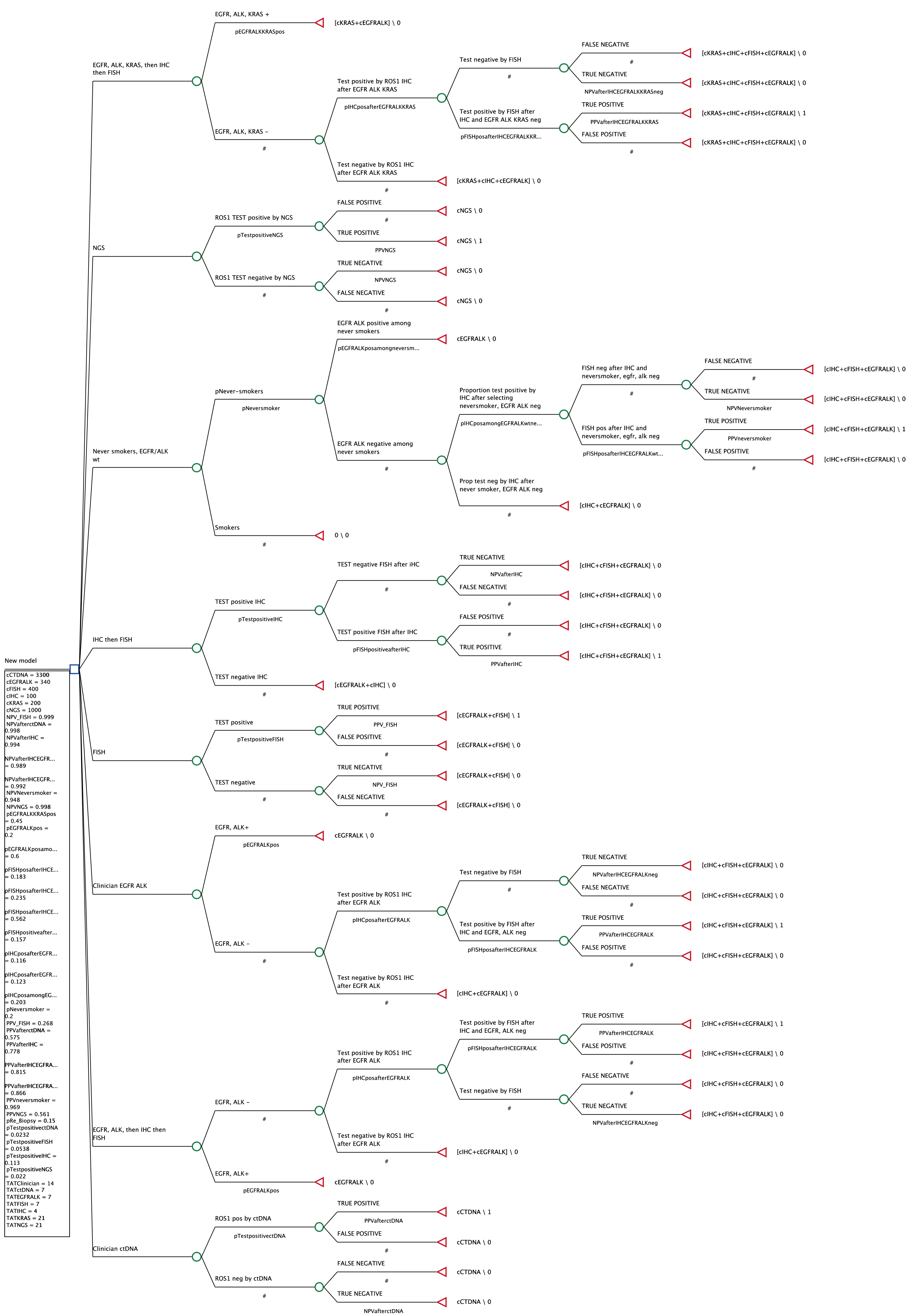

Supplement: Supplementary file 1 [file curroncol-28-00284-s001.zip › Supplementary Figure 1.pdf]
